# An Account of Diseases in an Eastern District of London, from May 20, to June 20, 1808

**Published:** 1808-07

**Authors:** 


*An Account of Diseases in an Eastern District of London, from May 20, to June 20, 1808.*

| | | | |
|---|---|---|---|
| ACUTE DISEASES. | | Vomitus - - - - | 2 |
| Typhus - - - - | 2 | Enterodynia - - - | 3 |
| Ephemera - - - | 5 | Dysuria - - - - | 2 |
| Rubeola - - - - | 3 | Fluor albus - - - | 5 |
| Pleuritis - - - | 2 | Menorrhagia - - - | 4 |
| Rheumatismus Acutus | 3 | Rheumatismus Chronicus | 10 |
| CHRONIC DISEASES. | | PUERPERAL DISEASES. | |
| Tussis - - - - | 9 | Menorrhagia Lochialis | 4 |
| Tussis cum Dyspnœa | 12 | Dolores post partum | 7 |
| Hydrothorax - - - | 5 | Rhagas Papillæ - - | 3 |
| Anasarca - - - | 7 | Hæmorrhois - - - | 2 |
| Ascites - - - - | 5 | INFANTILE DISEASES. | |
| Cephalalgia - - - | 7 | Apthhæ - - - - | 7 |
| Paralysis - - - | 2 | Ophthalmia - - - | 3 |
| Vertigo - - - - | 5 | Vermes - - - - | 2 |
| Dyspepsia - - - | 5 | Herpes - - - - | 4 |

There have been several cases of Aphthæ amongst children, some of which have proved troublesome and obstinate. This disease is so familiar to those who have the care of new-born infants, that every nurse supposes herself capable of curing it. It is sometimes a very slight disease, appearing only in a few white specks in different parts of the mouth, and disappearing without the assistance of any medicine. It is often, however, protracted by an injudicious attempt to cure it.

Rubbing the mouth with a rag-mop, as the nurses terms it, must be painful to the child; and if it removes the present appearance of the white specks, it is likely to be succeeded by another crop, which may be more troublesome and more difficult to cure.

In one of the cases referred to in the list, this complaint occurred to a child that had been much indisposed for some time, and it seemed to prove critical, as it was succeeded by a more confirmed state of health than it had before enjoyed.

This disease is in general most effectually removed by keeping the bowels moderately open by the use of some gently laxative medicine.

